# Genomic profiling of WRKY transcription factors and functional analysis of CcWRKY7, CcWRKY29, and CcWRKY32 related to protoberberine alkaloids biosynthesis in *Coptis chinensis* Franch

**DOI:** 10.3389/fgene.2023.1151645

**Published:** 2023-03-23

**Authors:** Xiaoqiang Huang, An Jia, Tao Huang, Li Wang, Guohua Yang, Wanli Zhao

**Affiliations:** ^1^ Zhengzhou Key Laboratory of Antitumor Traditional Chinese Medicine Research, Medical College, Huanghe University of Science and Technology, Zhengzhou, China; ^2^ Shizuishan Hospital of Traditional Chinese Medicine, Shizuishan, China; ^3^ Jiangsu Key Laboratory for the Research and Utilization of Plant Resources, Institute of Botany, Jiangsu Province and Chinese Academy of Sciences (Nanjing Botanical Garden Memorial Sun Yat-Sen), Nanjing, China

**Keywords:** protoberberine alkaloids, berberine biosynthesis, Coptis chinensis, WRKY family, transcription factors

## Abstract

*Coptis chinensis* Franch. (Huanglian in Chinese) is an important economic crop with medicinal value. Its rhizome has been used as a traditional herbal medicine for thousands of years in Asia. Protoberberine alkaloids, as the main bioactive component of *Coptis chinensis*, have a series of pharmacological activities. However, the protoberberine alkaloids content of *C. chinensis* is relatively low. Understanding the molecular mechanisms affecting the transcriptional regulation of protoberberine alkaloids would be crucial to increase their production *via* metabolic engineering. WRKY, one of the largest plant-specific gene families, regulates plant defense responses *via* the biosynthesis of specialized metabolites such as alkaloids. Totally, 41 WRKY transcription factors (TFs) related to protoberberine alkaloid biosynthesis were identified in the *C. chinensis* genome and classified into three groups based on phylogenetic and conserved motif analyses. Three WRKY genes (*CcWRKY7*, *CcWRKY29*, and *CcWRKY32*) may regulate protoberberine alkaloid biosynthesis, as suggested by gene-specific expression patterns, metabolic pathways, phylogenetic, and dual-luciferase analysis. Furthermore, the CcWRKY7, CcWRKY29, and CcWRKY32 proteins were specifically detected in the nucleus *via* subcellular localization. This study provides a basis for understanding the regulatory mechanisms of protoberberine alkaloid biosynthesis and valuable information for breeding *C. chinensis* varieties.

## 1 Introduction

*Coptis chinensis* belongs to the Ranunculaceae family and is primarily distributed throughout Southern China, specifically in the Sichuan, Hubei, Hunan, and Guizhou Provinces. The rhizomes of *Coptis chinensis* (“Huanglian” in Chinese) are a traditional herbal medicine used to treat infectious and inflammatory diseases (Tseng et al., 2020; Yang et al., 2021; Tseng et al., 2022; Yan et al., 2023). The primary active ingredients in *C. chinensis* include protoberberine-type alkaloids such as berberine, palmatine, tetrahydropalmatine, coptisine, jatrorrhizine, and columbamine (Liu et al., 2016; Tan et al., 2016; Wu et al., 2019; Li et al., 2021). With the gradual development of society, people pay more attention to their health status, increasing the demand for *C. chinensis* in different degrees. In recent years, the amount of *C. chinensis* has continually increasingly reduced due to over-excavation or exploitation of this Huanglian. Synthetic biology provides a new approach to the acquisition of active ingredients in natural products, which is green and efficient characteristics. The biosynthesis of terpenoids, flavonoids and alkaloids has recently been achieved *via* synthetic biology (Paddon et al., 2013; Nakagawa et al., 2016; Wu et al., 2022). Therefore, further investigation into the underlying mechanisms of protoberberine alkaloid biosynthesis using a synthetic biology approach to increase production is the key to achieving industrialized production.

The WRKY transcription factors (TFs) family play a significant role in plant stress response, including regulation of specialized metabolite biosynthesis (Schluttenhofer and Yuan, 2015; Vashisht et al., 2016; Barco and Clay, 2019; Zhang et al., 2020). The WRKY family contains about 60 amino acid binding domains and covers Classes I-III according to the number of WRKY domains and the variety of zinc finger motifs (Rushton et al., 2010; Hussain et al., 2019). Several WRKY TFs have been shown to regulate terpenoid and alkaloid biosynthesis functionally (Li et al., 2019; Hao et al., 2021; Yamada et al., 2021). For example, the TF, *AaWRKY1,* positively regulates the biosynthesis of artemisinin *via* promoting the expression of the genes *DBR2*, *CYP71AV1*, and *ADS* in *Artemisia annua* (Han et al., 2014). *CjWRKY1* regulates the biosynthesis of berberine in *Coptis japonica* (Yamada and Sato, 2016), and *PsWRKY1* regulates the biosynthesis of morphine in *Papaver somniferum* (Mishra et al., 2013). These data suggest that potential WRKY genes were functionally diverse among protoberberine-producing medicinal plant species and secondarily obtained additional functions associated with protoberberine biosynthesis in *C. chinensis*.

This research investigated the WRKY family genes in the *C. chinensis* genome *via* bioinformatics. The conserved domains, expression profiles of the CcWRKY genes in various tissues, and the main components of *C. chinensis* were analyzed. CcWRKY7, CcWRKY29, and CcWRKY32 proteins were detected using subcellular localization. Finally, three CcWRKY genes were identified that could be involved in berberine biosynthesis, providing the foundation for a more in-depth regulatory study.

## 2 Results and discussions

### 2.1 Genome-wide identification, conserved motifs composition, and classification of the CcWRKY family in *C. chinensis*

**FIGURE 1:**
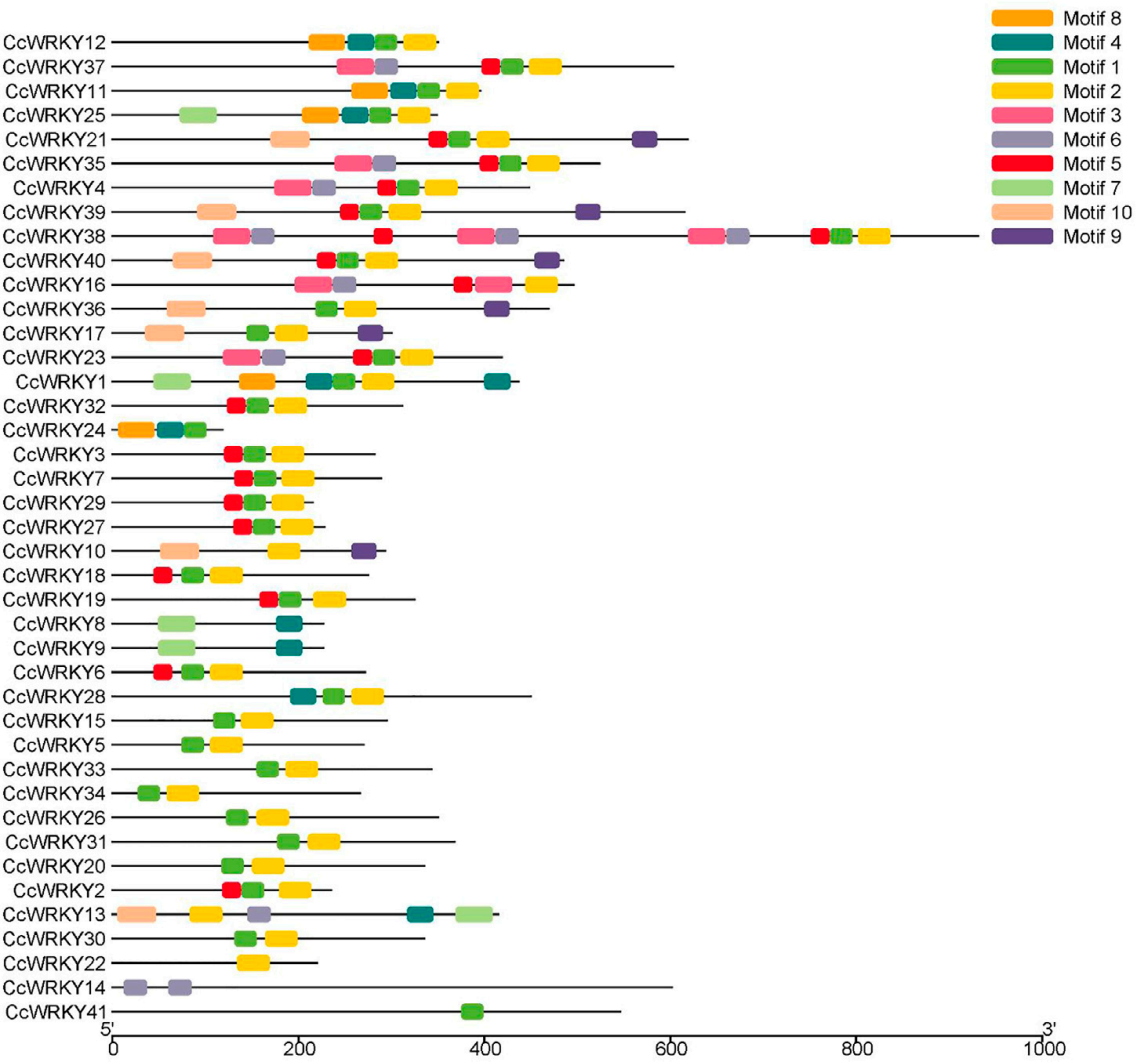
Schematic diagram of CcWRKYs conserved motifs composition.

Gene annotation information using a typical WRKY domain was used to search the *C. chinensis* genome to identify the WRKY genes. 41 WRKY genes were identified from *C. chinensis* following the removal of incomplete sequences. The WRKY domain was further confirmed using TBtools software (Chen et al., 2020). The 41 putative CcWRKY genes were designated as *CcWRKY1* to *CcWRKY41*. Table 1 contains the complete data for CcWRKY genes, including gene names, gene IDs, protein lengths, molecular weights (MW), and isoelectric points (pI). The protein lengths ranged from 118 to 931 amino acids (aa), the MW ranged from 13,358.29 to 103,620.60 Da, and the pI varied from 4.96 to 10.17. In order to further comprehend the conservation and diversity of the proteins, 10 motifs were identified by analyzing the CcWRKYs conserved motifs using the MEME program. Motif 1 and motif 2 were present in most genes (Figure 1) and most genes contained two or more motifs except CcWRKY22 and CcWRKY41. The same subgroups of CcWRKY members usually contained similar motifs (Supplementary Figure S1).

**TABLE 1:** Molecular characteristics of CcWRKYs in *C. chinensis*.

Gene name	IDs	Strand	Length (aa)	MW (Da)	pI
CcWRKY1	rna-gnl|WGS:JADFTS|Cch00001048-RA	**−**	437	46,963.00	9.67
CcWRKY2	rna-gnl|WGS:JADFTS|Cch00003415-RA	**−**	235	26,552.70	5.70
CcWRKY3	rna-gnl|WGS:JADFTS|Cch00004362-RA	**−**	282	31,368.59	7.11
CcWRKY4	rna-gnl|WGS:JADFTS|Cch00005344-RA	**−**	448	49,147.24	9.11
CcWRKY5	rna-gnl|WGS:JADFTS|Cch00007362-RA	**+**	270	30,808.14	4.96
CcWRKY6	rna-gnl|WGS:JADFTS|Cch00007778-RA	**−**	272	30,262.57	5.18
CcWRKY7	rna-gnl|WGS:JADFTS|Cch00008762-RA	**+**	289	32,737.47	6.45
CcWRKY8	rna-gnl|WGS:JADFTS|Cch00009983-RA	**−**	227	25,307.30	9.60
CcWRKY9	rna-gnl|WGS:JADFTS|Cch00032628-RA	**−**	279	31,161.97	9.21
CcWRKY10	rna-gnl|WGS:JADFTS|Cch00011599-RA	**+**	293	32,092.12	7.75
CcWRKY11	rna-gnl|WGS:JADFTS|Cch00013919-RA	**+**	396	44,079.00	9.82
CcWRKY12	rna-gnl|WGS:JADFTS|Cch00013922-RA	**−**	350	39,617.16	9.84
CcWRKY13	rna-gnl|WGS:JADFTS|Cch00014307-RA	**+**	415	46,387.62	9.55
CcWRKY14	rna-gnl|WGS:JADFTS|Cch00016152-RA	**−**	602	68,699.88	7.63
CcWRKY15	rna-gnl|WGS:JADFTS|Cch00016955-RA	**−**	295	33,373.95	5.83
CcWRKY16	rna-gnl|WGS:JADFTS|Cch00018185-RA	**+**	496	55,233.20	9.00
CcWRKY17	rna-gnl|WGS:JADFTS|Cch00020609-RA	+	300	33,307.39	5.22
CcWRKY18	rna-gnl|WGS:JADFTS|Cch00020783-RA	+	275	30,503.44	5.29
CcWRKY19	rna-gnl|WGS:JADFTS|Cch00021919-RA	**−**	325	36,262.16	7.75
CcWRKY20	rna-gnl|WGS:JADFTS|Cch00021997-RA	+	335	37,816.93	5.01
CcWRKY21	rna-gnl|WGS:JADFTS|Cch00022038-RA	**−**	619	67,530.02	6.38
CcWRKY22	rna-gnl|WGS:JADFTS|Cch00022824-RA	+	220	24,802.76	8.99
CcWRKY23	rna-gnl|WGS:JADFTS|Cch00024258-RA	+	419	46,437.71	9.03
CcWRKY24	rna-gnl|WGS:JADFTS|Cch00024520-RA	**−**	118	13,358.29	10.17
CcWRKY25	rna-gnl|WGS:JADFTS|Cch00025055-RA	+	349	38,552.49	9.40
CcWRKY26	rna-gnl|WGS:JADFTS|Cch00027653-RA	+	350	39,551.20	5.70
CcWRKY27	rna-gnl|WGS:JADFTS|Cch00028018-RA	+	228	26,341.58	8.86
CcWRKY28	rna-gnl|WGS:JADFTS|Cch00028868-RA	+	450	49,079.25	5.53
CcWRKY29	rna-gnl|WGS:JADFTS|Cch00028964-RA	+	215	24,487.06	8.50
CcWRKY30	rna-gnl|WGS:JADFTS|Cch00030369-RA	+	335	37,393.73	5.45
CcWRKY31	rna-gnl|WGS:JADFTS|Cch00030371-RA	**−**	368	40,557.41	5.56
CcWRKY32	rna-gnl|WGS:JADFTS|Cch00031519-RA	+	312	35,211.28	6.41
CcWRKY33	rna-gnl|WGS:JADFTS|Cch00031916-RA	+	343	38,437.85	5.28
CcWRKY34	rna-gnl|WGS:JADFTS|Cch00032508-RA	**−**	266	28,842.05	6.03
CcWRKY35	rna-gnl|WGS:JADFTS|Cch00032868-RA	+	524	57,308.57	8.61
CcWRKY36	rna-gnl|WGS:JADFTS|Cch00035395-RA	+	469	52,263.96	5.87
CcWRKY37	rna-gnl|WGS:JADFTS|Cch00036723-RA	+	603	66,493.17	5.58
CcWRKY38	rna-gnl|WGS:JADFTS|Cch00036724-RA	+	931	103,620.60	5.67
CcWRKY39	rna-gnl|WGS:JADFTS|Cch00037535-RA	+	615	66,542.42	6.02
CcWRKY40	rna-gnl|WGS:JADFTS|Cch00038035-RA	**−**	485	53,563.73	7.94
CcWRKY41	rna-gnl|WGS:JADFTS|Cch00040369-RA	**−**	546	61,190.49	9.86

**FIGURE 2:**
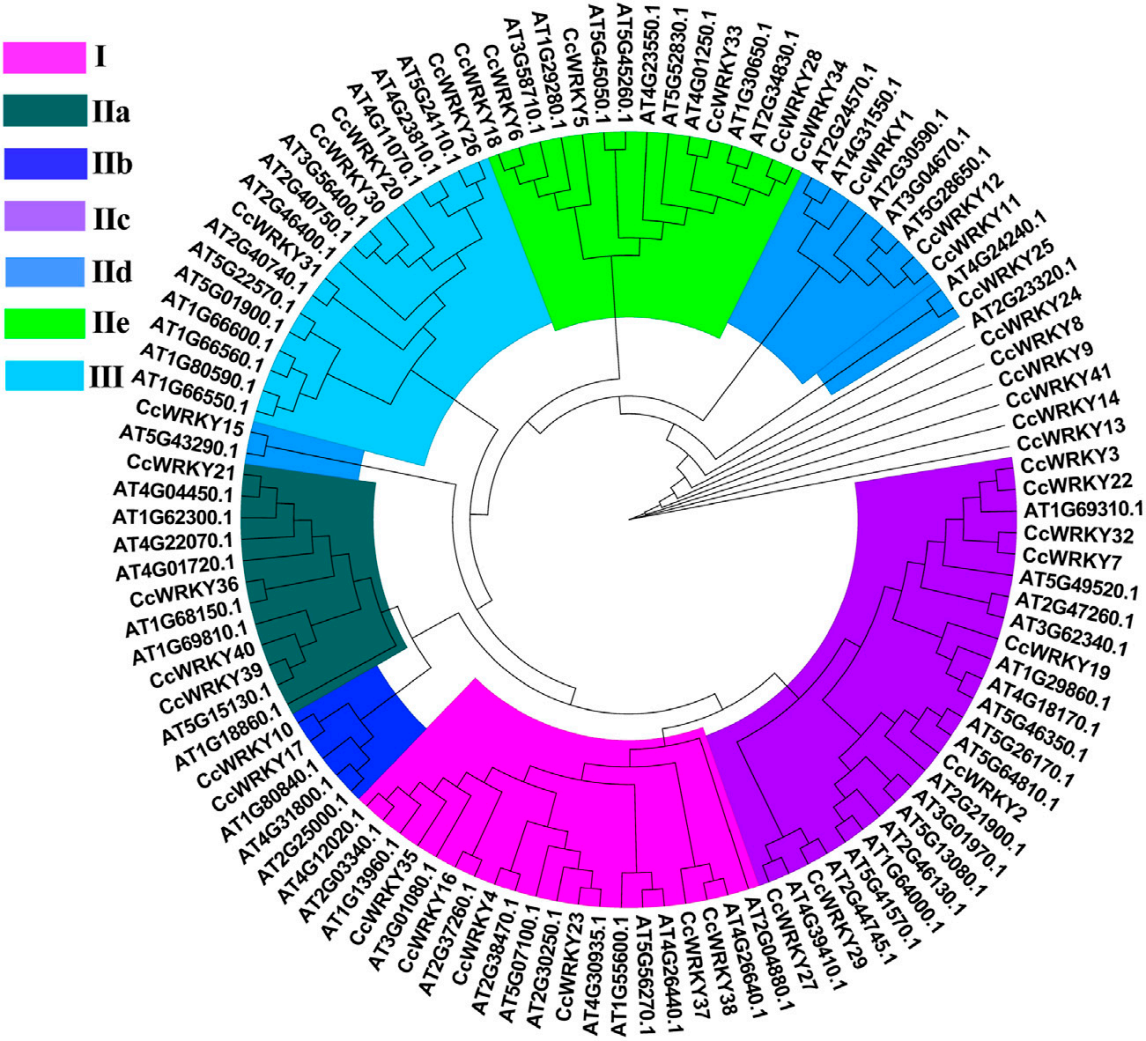
Phylogenetic relationships of CcWRKY proteins in *Coptis chinensis* and *A. thaliana*.

To classify the 41 CcWRKY proteins, an unrooted Maximum Likelihood (ML) phylogenetic tree was created using the CcWRKYs and *Arabidopsis thaliana* AtWRKYs proteins (Figure 2). According to the AtWRKYs classification, the CcWRKY proteins were separated into three groups (Group I-III). Group I had 6 members with two WRKY conserved domains (the N-terminal and C-terminal WRKY domains). Group II comprised twenty-five members, each harboring one WRKY domain (Supplementary Figure S1). According to the amino acid sequences, Group II was further categorized into subgroups IIa through IIe. Four CcWRKY proteins were classified in Group III, containing one WRKY domain. Group I and II had a C2H2 zinc finger motif, while Group III harbored the C2HC motif. Six CcWRKY proteins, including CcWRKY8, CcWRKY9, CcWRKY13, CcWRKY14, CcWRKY24, and CcWRKY41, did not cluster with any of the above Groups despite containing a WRKY domain.

### 2.2 Chromosomal locations

**FIGURE 3:**
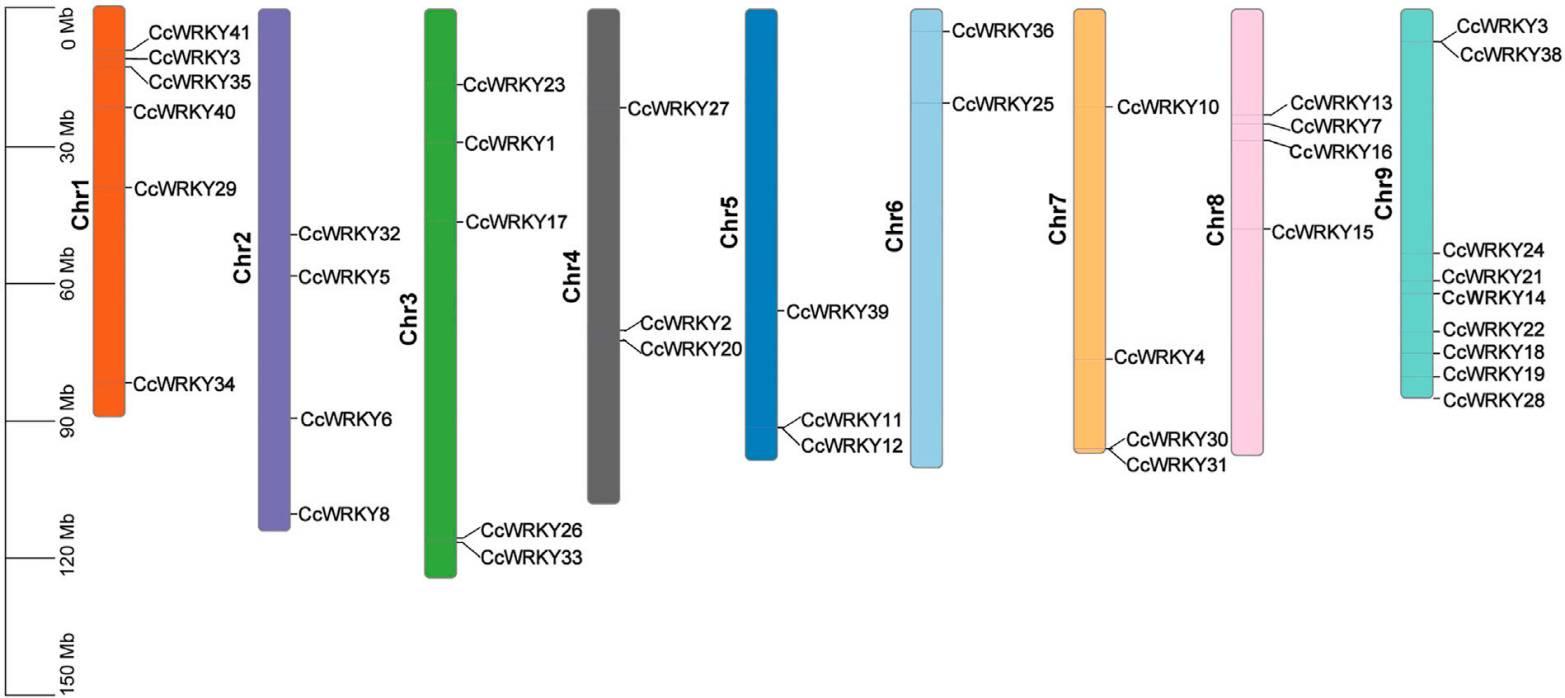
Chromosomal location of CcWRKY gene family in *C. chinensis*.

Thirty-nine CcWRKY genes were distributed on nine chromosomes, and two genes (CcWRKY9 and CcWRKY29) did not belong to any chromosome (Figure 3). Most of the CcWRKY genes were abundant on chromosome **9** (nine genes, 21.95%) and chromosome **1** (six genes, 14.63%), whereas they were only two WRKY genes on chromosome **6**. In addition, four CcWRKY genes were on chromosome **2**, five on chromosome **3**, and three on chromosome **4**. Three and four CcWRKYs were on chromosomes **5** and **7**, respectively, and four genes were identified on chromosome **8**. The uneven distribution of CcWRKY genes may be due to the variation in the sizes and structures of *C. chinensis* chromosomes.

### 2.3 Metabolic analysis

**FIGURE 4:**
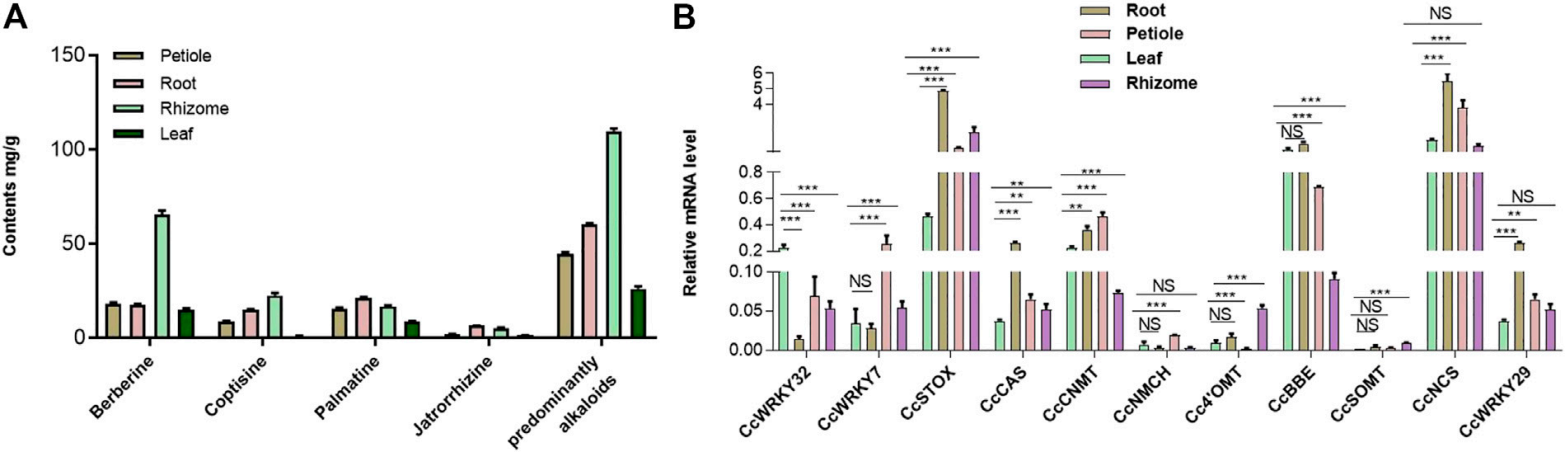
The main protoberberine alkaloids content of *C. chinensis* in different tissues and the expression profile of CcWRKY genes. **(A)** The main protoberberine alkaloid content of *Coptis chinensis* in different tissues. **(B)** Expression profile of CcWRKY genes and their candidate target genes involved in protoberberine alkaloids biosynthesis in different tissues including, root, petiole, leaf, and rhizome.

To investigate the tissue-specific accumulation patterns of alkaloids in *C. chinensis*, HPLC-QTOF-MS/MS was performed on samples, including the root, rhizome, petiole, and leaf tissues. Numerous protoberberine alkaloids found primarily in *C. chinensis* have been identified. These included significantly high levels of berberine, followed by jatrorrhizine, palmatine, and coptisine. Interestingly, the predominantly alkaloids and berberine content in tissues was as follows: rhizome > root > petiole > leaf (Figure 4A). However, palmatine and jatrorrhizine content was as follows: root > rhizome > petiole > leaf. These results suggest that the protoberberine biosynthetic enzymes may be tissue-specific.

### 2.4 Expression patterns of CcWRKY7, CcWRKY29 and CcWRKY32 in different tissues of in *C. chinensis*

The expression profiles of genes in different tissues can provide essential information for for screening functional genes. Previous research demonstrated that *CjWRKY1* overexpression in *Eschscholzia californica* cells strongly enhances benzylisoquinoline alkaloid (BIA) biosynthesis (Yamada et al., 2017; Yamada et al., 2021). Homology search using *CjWRKY1* amino acid sequences as queries against the genome database of *C. chinensis* revealed a similarity of *CjWRKY1* with the *CcWRKY7*, *CcWRKY29*, and *CcWRKY32*. The expression levels of these three genes in *C. chinensis* were analyzed by qRT–PCR. Furthermore, identifying the expression pattern of protoberberine biosynthetic genes can aid in the understanding of berberine biosynthesis regulation. Subsequently, *CcWRKY7*, *CcWRKY29*, *CcWRKY32*, and eight protoberberines biosynthetic genes (*CcNCS*, *Cc4′OMT*, *CcCNMT*, *CcNMCH*, *CcBBE*, *CcSOMT*, *CcSTOX*, and *CcCAS*) were detected in the root, rhizome, petiole, and leaf tissues. The expressions of *CcWRKY7*, *CcWRKY29*, and *CcWRKY32* were very similar to *CcCNMT* (the upstream genes in protoberberine biosynthesis). The transcription factor CcWRKY29 and the biosynthetic genes *CcBBE*, *CcCNMT*, *CcSTOX*, and *CcCAS*, that act on the downstream enzymes for the biosynthesis of predominant protoberberines (berberine and palmatine), were significantly overexpressed in root compared with other tissues (Figure 4B, Supplementary Figure S2).

### 2.5 Subcellular localization analysis of the CcWRKY7, CcWRKY29 and CcWRKY32

**FIGURE 5:**
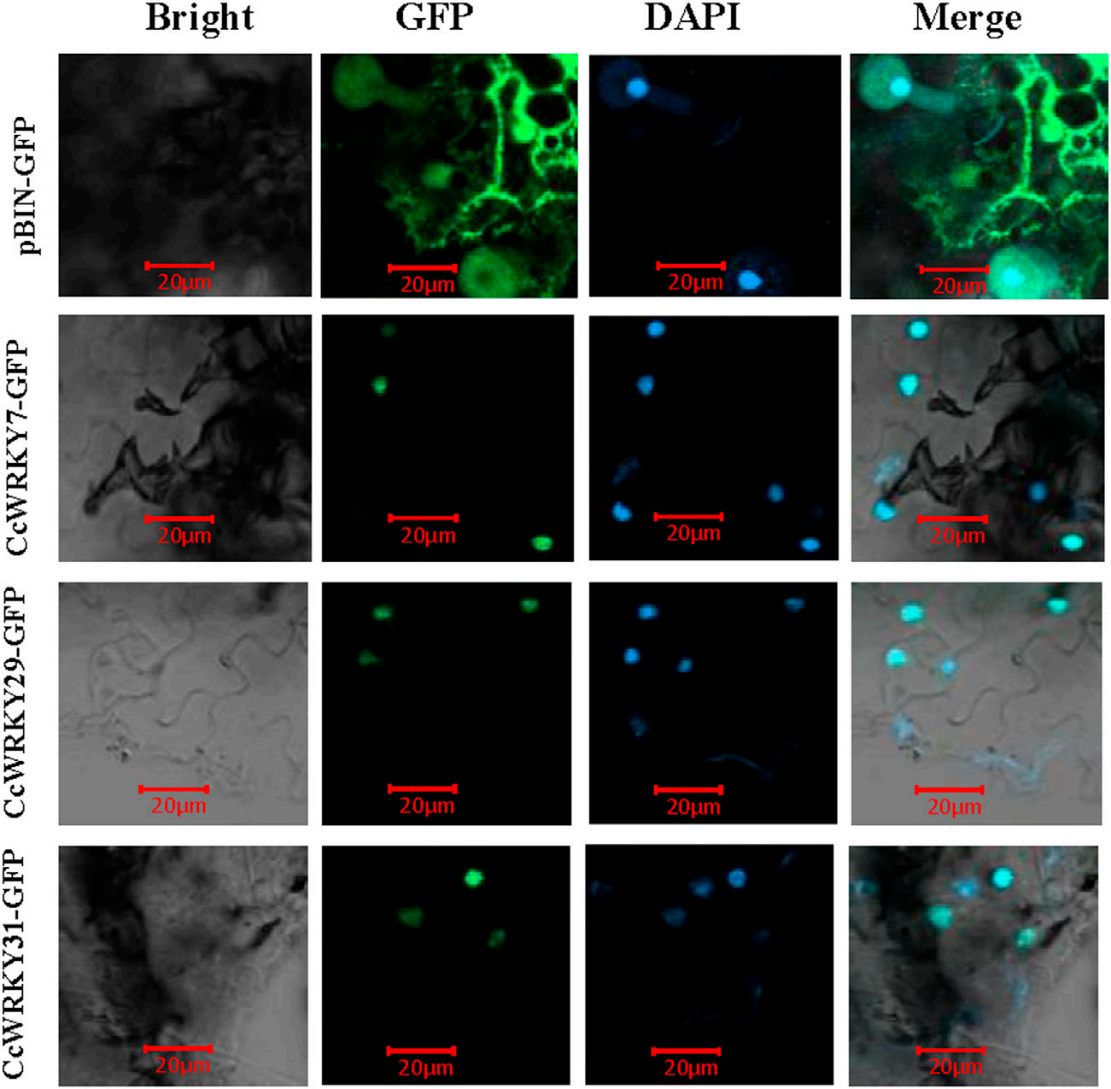
Subcellular localization of CcWRKY7, CcWRKY29 and CcWRKY32 proteins in *Nicotiana benthamiana* leaves. Laser scanning confocal microscope with wavelengths of 488 and 406 nm for GFP and DAPI, respectively.

To analyze the prediction results of CcWRKY7, CcWRKY29, and CcWRKY32 subcellular localization, full-length CDSs of these three genes (lacking the stop codon) were cloned into the pCAMBIA2300 vector (containing the 35S promoter and GFP). Transient transformation assays were done using *N. benthamiana* Domin leaves. The results demonstrated that the GFP fused CcWRKY7, CcWRKY29, and CcWRKY32 proteins were specifically expressed in the nucleus of the *Nicotiana benthamiana* leaf. In contrast, the GFP control protein was detected in various organelles (Figure 5). The nuclear localization results were consistent with the expected role of CcWRKYs as transcription factors.

### 2.6 CcWRKYs regulating protoberberine biosynthetic gene

**FIGURE 6:**
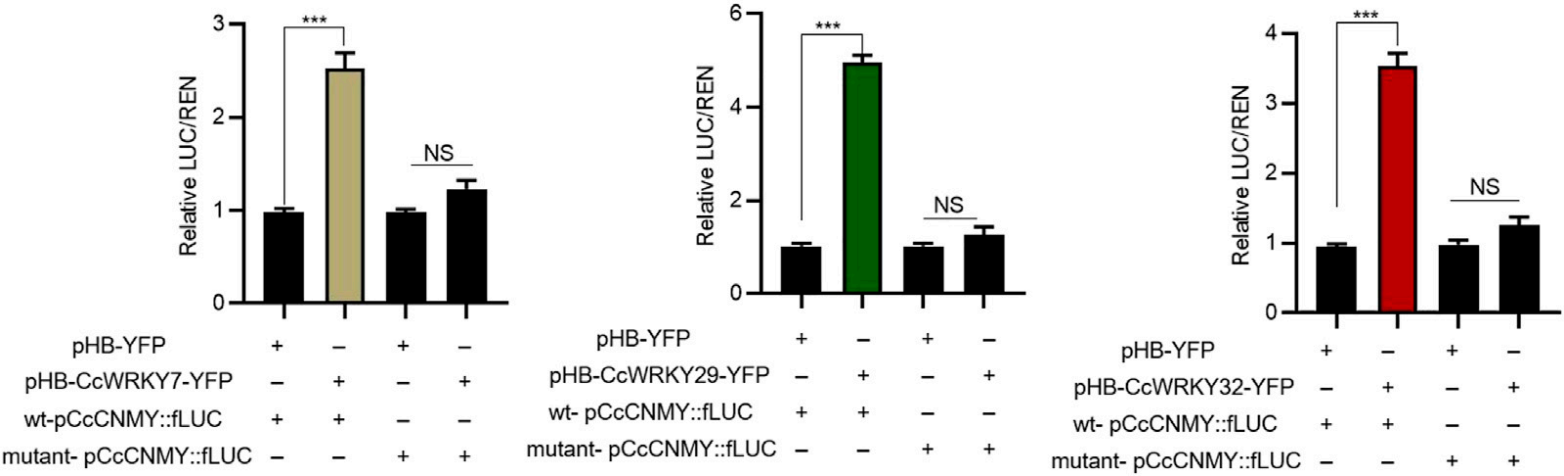
CcWRKY7, CcWRKY29, and CcWRKY32 activate the promoter of *CcCNMT*.

The candidate target genes promoter elements of CcWRKYs transcription factors analyzed. It was showed that protoberberines biosynthetic genes (CcNCS, Cc4′OMT, CcCNMT, and CcSOMT contained W-box element, necessary binding site of WRKY. This suggests that CcWRKY may play a role in regulating protoberberines biosynthesis by regulating the expression of above target genes.To further verify *CcWRKY7*, *CcWRKY29*, and *CcWRKY32* regulate the protoberberine biosynthetic gene, dual-luciferase assays were performed in *N. benthamiana* leaves. The results showed that CcWRKY7-pCcCNMT, CcWRKY29-pCcCNMT, and CcWRKY7-pCcCNMT significantly higher reporter activation than controls, respectively (Figure 6). Taken together, our results suggest that CcWRKY7, CcWRKY29, and CcWRKY32 can bind and activate the promoter of *CcCNMT*, respectively.

Previous studies reported that the medicinal plant *C. chinensis* is a diploid (2n = 18), and the genomic heterozygosity proportion is 0.77. The chromosome-level genome of *C. chinensis* was −958.20 Mb with a scaffold N50 of 4.53 Mb, and a contig N50 of 1.58 Mb (Chen DX. et al., 2021). A total of 34,109 protein-coding genes were identified in *C. chinensis* with an average CDS coding sequence length of 1031.26 bp. The genomic data of *C. chinensis* provides abundant gene resources for studying the biosynthetic pathway of berberine, especially the key enzyme genes and regulated transcription factors. Protoberberine alkaloids, the main ingredient of *C. chinensis*, have a series of biological activities (Qin et al., 2018; Chen Q. et al., 2021). As the demand for protoberberine alkaloids has grown in recent years, obtaining higher content of protoberberine alkaloids by controlling their biosynthetic pathway has attracted much attention. TFs can promote compound production by activating biosynthetic enzymes at the transcriptional level (Suttipanta et al., 2011). Therefore, this study investigated the WRKY TFs involved in regulating protoberberine alkaloid biosynthesis, and 41 WRKY genes were identified in the *C. chinensis* genome. Meanwhile, the conserved domains, the major components in *C. chinensis*, and expression profiles of the CcWRKY genes in different tissues were further analyzed. The phylogenetic, gene tissue-specific expression, dual-luciferase analysis suggested that three CcWRKY genes may regulate protoberberine alkaloid biosynthesis. The CcWRKY7, CcWRKY29, and CcWRKY32 proteins were specifically detected in the nucleus. The WRKY TFs are widely distributed throughout the plant kingdom, playing an essential role in plant growth and stress response (Liu et al., 2020). Moreover, developing the CcWRKY gene profile is useful for future morphogenesis and resistance formation studies in *C. chinensis*. Although we preliminarily found that three CcWRKYs may play a role in regulating berberine biosynthesis *via* the target gene *CcCNMT*, the molecular mechanism remains to be clarified, which is also our future focus.

## 3 Materials and methods

### 3.1 Plant material

The diploid cultivar of *Coptis chinensis* (two-year-old), which was originally collected from Mianyang, Sichuan. The plants were transferred to our laboratory greenhouse to acclimate for 2 weeks before the experiment began. The root, rhizome, petiole, and leaf of the *C. chinensis* were collected in three biological replicates. All samples were frozen in liquid nitrogen after incubating in RNAlater and then stored at −80°C for RNA extraction. Samples were freeze-dried under a vacuum and powdered ground for metabolic analysis.

### 3.2 Identification of CcWRKY genes in *C. chinensis*

The whole genome sequence of *C. chinensis* (Accession Number PRJNA649082) was downloaded from NCBI, and AtWRKY protein sequences from TAIR (https://www.arabidopsis.org) (Supplementary Text S1). Firstly, the CcWRKY protein sequences were queried using Auto BLAST and HMM to search for potential CcWRKY proteins. Secondly, the potential CcWRKY annotation information was compared with the Swiss-Port database, and then, candidate CcWRKY proteins were submitted to NCBI-CDD. Pfam database was used to verify whether candidate CcWRKY proteins contained the conserved domains. Next, EXPASy-ProtParam was used to predict the coding sequence (CDS) length, the isoelectric point and molecular weights of all CcWRKY proteins. The Plant-mPLo website was used to predict the subcellular localization.

### 3.3 Phylogenetic, conserved motifs and chromosomal locations analysis

Phylogenetic analysis was conducted using the Maximum Likelihood method (ML) with 1,000 bootstrap replicates (Jones-Taylor-Thornton model, site coverage cut off 95%). The conserved motifs were performed using Batch-Search and the TBtools software. To better understand the functions of CcWRKY proteins, the protein sequences were analyzed for conserved motifs using the MEME program (https://meme-suite.org/meme/). The CcWRKY gene distribution map was generated using the TBtools software.

### 3.4 Metabolic analysis

Dry powder ground samples (0.1 g) with 5 mL methanol were sonicated for 30 min. Primary components of *C. chinensis* were determined using HPLC-QTOF-MS/MS (Agilent Technologies) with a C_18_ column (Agilent, 2.7 μm, 4.6 mm × 100 mm). The column temperature was 30°C, and the flow rate was set to 0.25 mL/min with a 5 μL injection volume. The mobile phase consisted of phase A (water containing 0.1% formic acid) and phase B (acetonitrile) with the following gradient program: 10% B at 0–2 min, 10%–20% B at 2–10 min, 20% B at 10–13 min, 20%–30% B at 13–16 min, 30% B at 16–17 min, 90%–90% B at 17–20 min, 90% B at 20–22 min, 90%–10% B at 22–23 min, 10% B at 23–25 min. Mass spectra were acquired using an electrospray ionization source, and the acquisition parameters in the positive mode were set as follows: drying gas flow rate, 10.0 L/min; temperature, 350°C; fragmentor voltage, 130 V and nebulizer, 35 psig; OCT RFV, 750 V; capillary voltage, 3500 V; collision energy 20 eV; Mass range from *m/z* 100 to 1700.

### 3.5 RNA extraction and quantitative real-time PCR analysis

Total RNA was extracted with TRIzol reagent (Sigma-Aldrich), and cDNA was synthesized by reverse transcription with HiScript qRT SuperMix (Vazyme, China). QRT-PCR assays were performed with a LC480 Light Cycler (Roche, Germany). The Ccβ-actin gene from *C. chinensis* was used as an internal standard to calculate relative fold differences based on the comparative cycle threshold values. The RT-qPCR procedure was performed on the following parameters: 95°C for 10 min; 40 cycles of 95°C for 15 s; 60°C for 60 s. All primers of the qRT–PCR are listed in Supplementary Table S1.

### 3.6 Subcellular localization analysis

The CcWRKYs CDS were constructed into a transient expression pCAMBIA2300 vector with a GFP fluorescent label and a CaMV 35S promoter. All amplification primers for plasmid construction were shown in Supplementary Table S1. The recombinant plasmid was transferred to *Agrobacterium* MSU440 *via* the conventional freezing-thawing method. Next, empty pCAMBIA2300-GFP or pCAMBIA2300-CcWRKY-GFP MSU440 were instantly infiltrated into 4-week-old *N. benthamiana* leaves with expression buffer (10 mM MgCl2, 10 mM MES pH 5.6, 100 µM acetosyringone). After the infiltrated *N. benthamiana* was in the dark for 24 h and then in low light for another 24 h, the leaf samples were detected using a Laser scanning confocal microscope (Zeiss LSM900).

### 3.7 LUC assays

The reporter constructs wt-pCcCNMT:fLUC and mutant-pCcCNMT:fLUC were obtained by inserting the promoter of CcCNMT and CcCNMT promoter with mutant W-box into the pGreenII 0800-LUC vector, to drive expression of the firefly luciferase gene. The Renilla luciferase (CaMV 35S promoter-driven) was used as an internal standard. The assembled vectors were co-transformed with the helper plasmid pSoup19 into *A. tumefaciens* strain EHA105. The *A. tumefaciens* strain EHA105 harboring pHBCcWRKY-YFP and pHB-YFP were used as the effector and the negative control, respectively. The reporter strains containing wt-pCcCNMT:fLUC or mutant-pCcCNMT:fLUC were mixed with effector strains harboring either pHBCcWRKY-YFP or pHB-YFP (a ratio of 1:1). The *A. tumefaciens* suspension was infiltrated into *N. benthamiana* leaves, while negative controls were infiltrated into the opposite parts of the same leaves (Hao et al., 2019). Leaves were collected after 48 h in the dark, and luciferase activities were detected using the Dual-Luciferase Reporter Assay System. All experiments were performed with three biological transfections.

## 4 Conclusion

This study identified 41 CcWRKY genes in the genome of *C. chinensis* and classified them into Groups I, II, and III based on phylogenetic relationships, motif patterns, and gene structure analysis. Phylogenetic, gene expression pattern, qRT-PCR, and dual-luciferase analysis suggest that *CcWRKY7*, *CcWRKY29*, and *CcWRKY32* candidate genes may be involved in regulating the protoberberine biosynthesis. CcWRKY7, CcWRKY29, and CcWRKY32 proteins were specifically detected in the nucleus. The information collected in this study provides a basis for further exploration regulation of berberine biosynthesis and may increase the production of valuable protoberberine in metabolic engineering.

## Data Availability

The datasets presented in this study can be found in online repositories. The names of the repository/repositories and accession number(s) can be found in the article/Supplementary Material.
