# Supplementary material for: Genomic profiling of WRKY transcription factors and functional analysis of CcWRKY7, CcWRKY29, and CcWRKY32 related to protoberberine alkaloids biosynthesis in Coptis chinensis Franch
Source: Front Genet. 2023 Mar 23;14:1151645. doi: 10.3389/fgene.2023.1151645 (PMC10076542; doi:10.3389/fgene.2023.1151645)
Supplement: Supplementary file 1 [file DataSheet1.ZIP › Supplementary Files/Supplementary Material.docx]

Table S1 The primers used in this study

|  | Primer name | Sequence (5’ to 3’) | Primer name | Sequence (5’ to 3’) |
| --- | --- | --- | --- | --- |
|  | CcWRKY7-F | ttctgcaggagctcggtaccATGGAG  ATGGCAAATTCAGTGG | CcWRKY7-R | cccttgctcaccatggatccTTCCTTCAC  TTGCTTACTCGAAGG |
|  | CcWRKY32-F | ttctgcaggagctcggtaccATGATG  GACAAGGTCAAGAAGGA | CcWRKY32-R | cccttgctcaccatggatccCTCGTCTTT  CCGAACATCTGATG |
| Primes for cloning | CcWRKY29-F | ttctgcaggagctcggtaccATGGAA  TCAGACCCAAGAGCTTC | CcWRKY29-R | cccttgctcaccatggatccGAATGAAT  TAAAGCCTTCATGTTCA |
|  | Vector-F | GGATCCATGGTGAGCAAGGG | Vector-R | GGTACCGAGCTCCTGCAGAA |
|  | Ccβ-Actin-q-F | GTCACACCGTCCCCATTTA | Ccβ-Actin-q-R | GTCACGGACGATTTCTCGTT |
|  | CcNCS-q-F | TCAATGGACGACCGCTACTC | CcNCS-q-R | AAATGCAAGCCCAACTCAGG |
|  | CcCNMT-q-F | GTGGAAGTCAAATTGGCAGA | CcCNMT-q-R | AAAAGAACGACGCAGATGGT |
|  | CcNMCH-q-F | GAAGAGCAGTGGCAGAGGTT | CcNMCH-q-R | TAGCAATGGTGTTGGAGGGT |
|  | Cc4'OMT-q-F | TTCAAGGAATGCGAAGAGGA | Cc4'OMT-q-R | ATTGGCAATGACATGAGGGA |
|  | CcBBE-q-F | CGGCTGGCTATTGCCCTACT | CcBBE-q-R | TCTTCACCCATTGATTTACG |
|  | CcSOMT-q-F | TGGTTCCACTATTGCTTTTG | CcSOMT-q-R | ATTACCGTTCCATTCTCAGG |
|  | CcCAS-q-F | CAAAAAAGCCGAGAGAGAGG | CcCAS-q-R | ATCAAGTAAGCGAGTGCCCA |
|  | CcSTOX-q-F | GGTGCAAACAGGGGCTACTA | CcSTOX-q-R | CCATTCACATCAACCAAACG |
|  | CcWRKY1-q-F | AGCAGCAACAGCAGGA | CcWRKY1-q-R | CACGACGAAAGCGAGC |
|  | CcWRKY2-q-F | CCCACCACAGACACAC | CcWRKY2-q-R | TCTTCTTCGGAACCAG |
|  | CcWRKY3-q-F | ATCAGATTGTTTGGCG | CcWRKY3-q-R | ACGGTTTATCAGGGGT |
|  | CcWRKY4-q-F | TCCTTCTGTTTCTCCT | CcWRKY4-q-R | GCATCGTTACTTTGAG |
|  | CcWRKY5-q-F | CTGATAAGACTGTGGT | CcWRKY5-q-R | TCTGATGTGTAGGTGA |
|  | CcWRKY6-q-F | CACCTACTCCATCTCC | CcWRKY6-q-R | TTAGTCGGCTTCTTTC |
|  | CcWRKY7-q-F | CTCCACCTCAACCACTATCT | CcWRKY7-q-R | GCTTTTTGTCCGTACTTTCT |
|  | CcWRKY8-q-F | TGGAGACTGAAATGGA | CcWRKY8-q-R | GGAGAACTGAACGAAA |
|  | CcWRKY9-q-F | TGCCGTCTTCTTCTAC | CcWRKY9-q-R | ATTTTATCCTCTTGCG |
|  | CcWRKY10-q-F | ACGAAGAATCAAGCAA | CcWRKY10-q-R | TGAAGAACCAACCAAG |
|  | CcWRKY11-q-F | GGAAAATGTGGTAGCA | CcWRKY11-q-R | ACTCATCAGGGGGTAT |
|  | CcWRKY12-q-F | TGGAAGTAGTGGTAAA | CcWRKY12-q-R | ATCCTAGAATGGTTGT |
|  | CcWRKY13-q-F | ACTGTAGGAGAGGGCA | CcWRKY13-q-R | TTGTTGGTGGGAAGAC |
|  | CcWRKY14-q-F | AGATGGACTGTAGGTA | CcWRKY14-q-R | TTTGAGAATGTGTTTG |
|  | CcWRKY15-q-F | GATGGAGATAACAGAG | CcWRKY15-q-R | CAATACAAAGAAAGGA |
| Primers for QPCR | CcWRKY16-q-F | AAGGATGACCGTTTGA | CcWRKY16-q-R | GAAGGAGCTGCTGAAT |
|  | CcWRKY17-q-F | AAATCGCCTTGTAGTG | CcWRKY17-q-R | GTTCGGATGGTTGTGT |
|  | CcWRKY18-q-F | CGGATAATAGTGGTGA | CcWRKY18-q-R | CTTGTTTTCTTGCTGG |
|  | CcWRKY19-q-F | TATTTCTTCGTCGTCC | CcWRKY19-q-R | TTTCTTCTTGGGTTTG |
|  | CcWRKY20-q-F | AAAGGAGAACAATGAC | CcWRKY20-q-R | ACTTAGCACCAAGAAT |
|  | CcWRKY21-q-F | CCCAAACCAAAACCTG | CcWRKY21-q-R | TGTATCCGCCAATGAC |
|  | CcWRKY22-q-F | TCTTTATGGCTTAGGC | CcWRKY22-q-R | TGAAGTGAACATCGGA |
|  | CcWRKY23-q-F | ACATCTGCTGGTTGCC | CcWRKY23-q-R | TCATTGGGGTTTGCTA |
|  | CcWRKY24-q-F | ACTCTTTCCAGTTGAT | CcWRKY24-q-R | CACTCTTAGTTTCCTT |
|  | CcWRKY25-q-F | CAACAACTACAGGAAC | CcWRKY25-q-R | CTAATAAGGAAATGAC |
|  | CcWRKY26-q-F | TTGGAGGAAGTATGGA | CcWRKY26-q-R | TTTTGTTTGGTCTGGT |
|  | CcWRKY27-q-F | AACTTGACGACCTTCC | CcWRKY27-q-R | CATCTTTGTGCTTTGG |
|  | CcWRKY28-q-F | GTAGTAACGAGGGAGT | CcWRKY28-q-R | ATGTTTGAGAAAGGAT |
|  | CcWRKY29-q-F | ATGGAATCAGACCCAA  GAGCTTCCA | CcWRKY29-q-R | TGGAAGCTCTTGGGTC  TGATTCCAT |
|  | CcWRKY30-q-F | TGCTCTACTGTGTCGG | CcWRKY30-q-R | AGTGGCTTTTTGTTTT |
|  | CcWRKY31-q-F | TTGAAGCAAATCTCCC | CcWRKY31-q-R | GCCCTTGGTTCTAATG |
|  | CcWRKY32-q-F | AAAGCAACAGAGGGA  GCCAA | CcWRKY32-q-R | ACACACCAAAGCAAG  CCGAA |
|  | CcWRKY33-q-F | TTTTTTCTCTTTTCCC | CcWRKY33-q-R | GCTGCTCTTGTTTCAT |
|  | CcWRKY34-q-F | TCAACACGAACACAAC | CcWRKY34-q-R | TACTTCACTCACTGCC |
|  | CcWRKY35-q-F | CAAAAGATGGTGGACA | CcWRKY35-q-R | TTGAAACAGAAGGTGC |
|  | CcWRKY36-q-F | ACAATATACACCTCCT | CcWRKY36-q-R | TTATCTACATCTCCCT |
|  | CcWRKY37-q-F | ATAAAGAAACAAGCCC | CcWRKY37-q-R | GTGGAAACTCACAACC |
|  | CcWRKY38-q-F | CCTGATAGATTTTGGG | CcWRKY38-q-R | TATATGACTGCTTGGC |
|  | CcWRKY39-q-F | GACGAAAAGAGGGTTA | CcWRKY39-q-R | TGGTTAGGTGAAGGAG |
|  | CcWRKY40-q-F | GAAAGATTGAAGACAC | CcWRKY40-q-R | CAAACTAAGAGAAAAG |
|  | CcWRKY41-q-F | AATGGACCAATAAAGC | CcWRKY41-q-R | CTCAGGTGGGAACTCT |


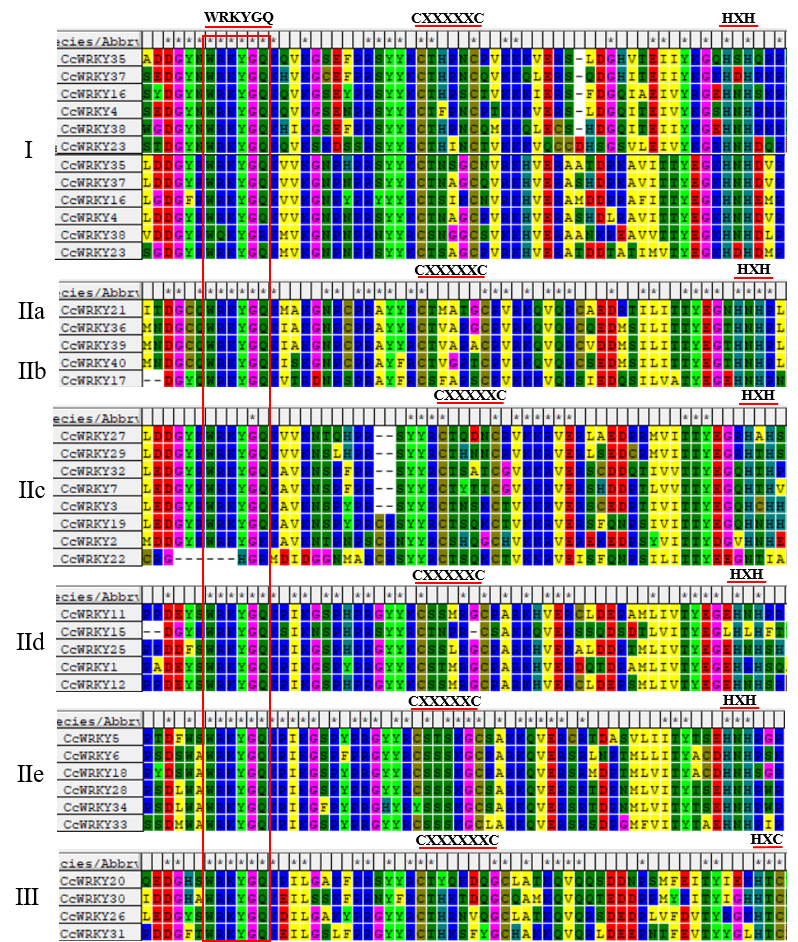


Fig. S1. Comparison of deduced amino acid sequences of the WRKY domains from CcWRKYs.


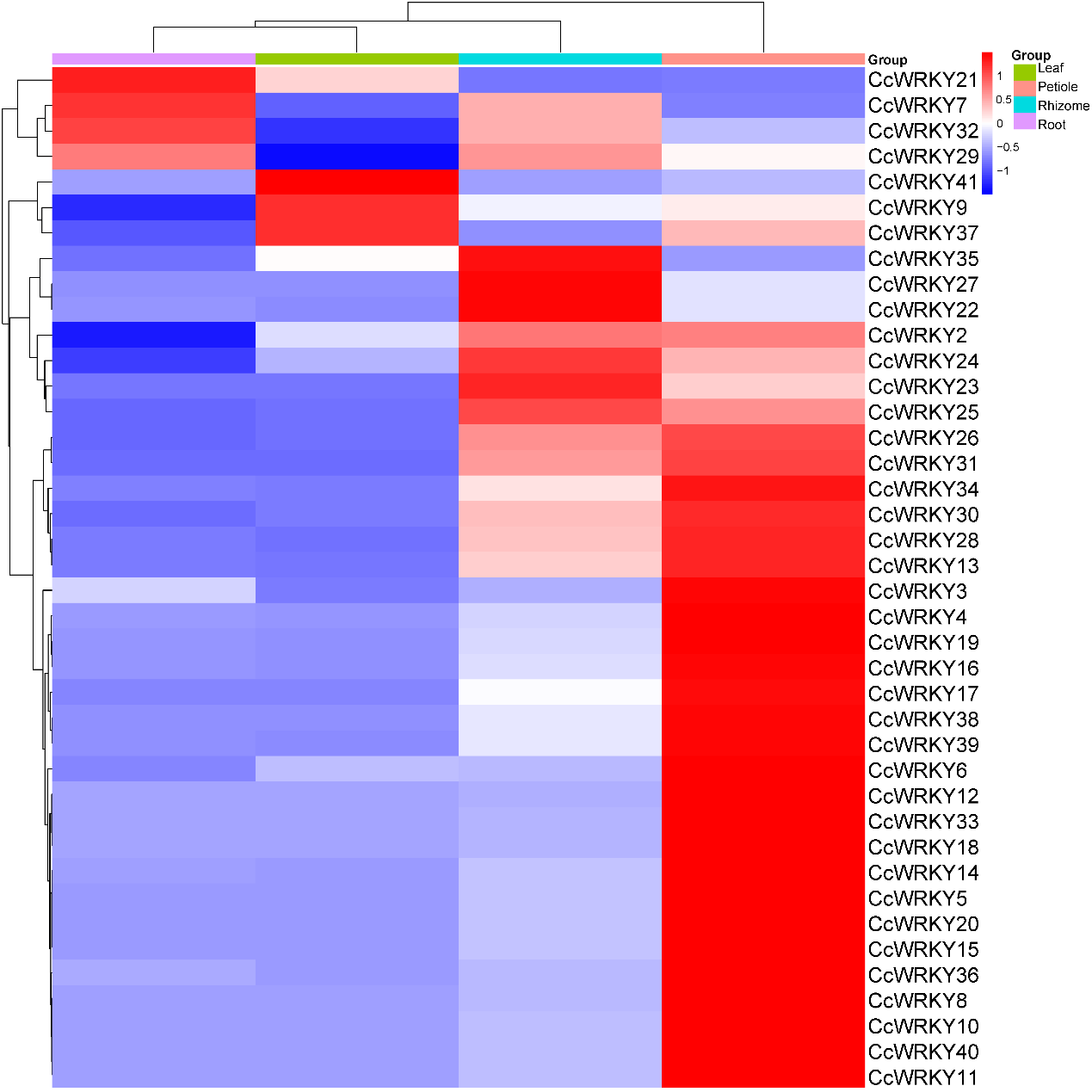


Fig. S2. The CcWRKY expression genes in different tissues of *C. chinensis*.
